# EMPOWERING FRAIL OLDER ADULTS THROUGH DIGITAL LITERACY: A THEORY AND VALUES-BASED INTERVENTION

**DOI:** 10.1093/geroni/igae098.1276

**Published:** 2024-12-31

**Authors:** Melissa Hladek, Olivia Rubio, Samantha Curriero, Deidra Crews, Mara McAdams DeMarco, Daniel Brennan, Qian-Li Xue, Sarah Szanton

**Affiliations:** Johns Hopkins University, Baltimore, Maryland, United States; The Pennsylvania State University, University Park, Pennsylvania, United States; New York University, New York, New York, United States; Johns Hopkins University, Baltimore, Maryland, United States; New York University, New York, New York, United States; Johns Hopkins University School of Medicine, Baltimore, Maryland, United States; Johns Hopkins University, Baltimore, Maryland, United States; Johns Hopkins University, Baltimore, Maryland, United States

## Abstract

Frailty is an age-associated syndrome heightening vulnerability to stressors. Mobility limitations common among individuals with frailty can impede access to physical resources and can lead to social isolation, detrimentally affecting mental and physical health. Digital literacy offers access to health information access, assistive and remote monitoring technologies and social engagement. Each are vital for people with frailty because they can enhance independence, cognition and quality of life. Despite the potential of digital literacy, internet access and digital health resources remain underutilized with older adults, especially among older adults with lower incomes and education levels. We designed a theory and values-based person-directed intervention to address digital literacy for older adults, aiming to recognize and leverage the motivational factors driving their desire to learn new skills. This is a 20 person one-arm feasibility and acceptability pilot trial enrolling participants with varying levels of frailty and digital literacy. The study is comprised of 4-6 in-home biweekly visits over the course of 8-12 weeks that use the older adults’ own values and goals to co-develop action plans that matter to the participant with the primary outcome of improvements in digital literacy. We hypothesize creating a digital literacy intervention that is person-directed will be feasible and acceptable to older adults and will lead to improvements in digital literacy, self-efficacy and quality of life.

